# Voltammetry Prediction and Electrochemical Analysis of Carbon Material from “Salt-In-Water” to “Water-In-Salt”

**DOI:** 10.1021/acs.analchem.4c04764

**Published:** 2025-01-31

**Authors:** Sukanlaya Kornnum, Praeploy Chomkhuntod, Nick Schwaiger, Kanwara Limcharoen, Krittapong Deshsorn, Kulpavee Jitapunkul, Pawin Iamprasertkun

**Affiliations:** † School of Bio-Chemical Engineering and Technology, Sirindhorn International Institute of Technology, Thammasat University, Pathum Thani 12120, Thailand; ‡ Research Unit in Sustainable Electrochemical Intelligent, Thammasat University, Pathum Thani 12120, Thailand; § Department of Chemical Engineering, Faculty of Engineering, Kasetsart University, Bangkok 10900, Thailand

## Abstract

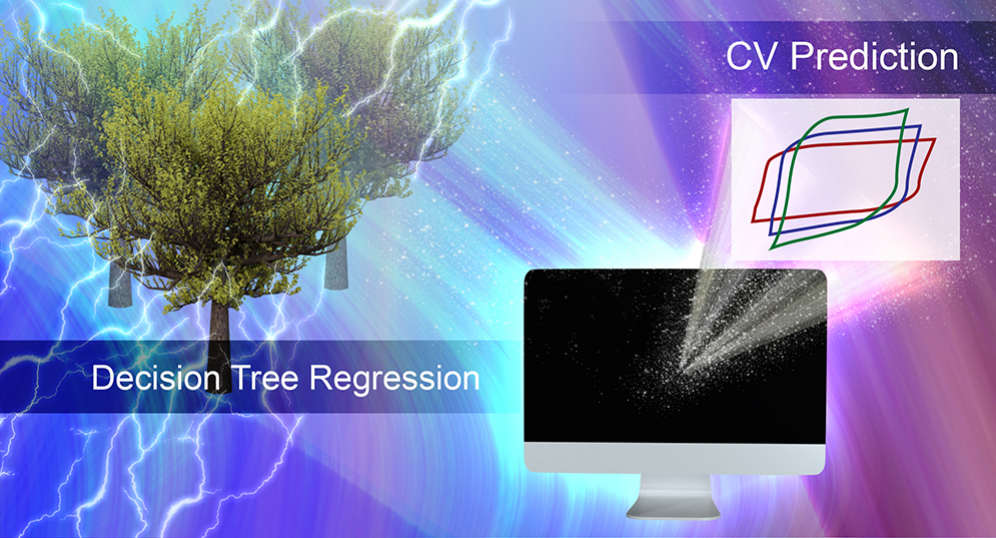

Cyclic voltammetry (CV) is a standard method for assessing electrochemical properties in the electrochemical cells, typically in conventional aqueous contexts like 1 *m* solutions (“salt-in-water”). However, recent advancements have extended electrochemistry into superconcentrated regimes, such as “water-in-salt” solutions with concentrations above 10 to 20 *m*, which require large amounts of salt for experiments. To address this, machine learning (ML) has been applied, coupled with in-house data collection using lithium bis(trifluoromethanesulfonyl)imide (LiTFSI) electrolytes. This work demonstrates the electrochemistry of YEC-8B in LiTFSI, given their broad potential window of up to 3.0 V across concentrations from 1 to 20 *m*. The CV profiles were divided into two models: the upper curve for charging and the lower curve for discharging. Data were normalized and segmented by percentiles, and a decision tree model was developed to predict outputs based on input parameters like LiTFSI concentration, scan rates, and potential window. The model predicted nine target variables with a mean absolute percentage error of approximately 2% for both the upper and the lower CV profile curves. Trapezoidal rule was then used to calculate the system’s capacitance. Additionally, tests showed a 75% accuracy in predicting the potential window and a suitable scan rate. Overall, the model effectively demonstrated the relationship between “water-in-salt” electrolytes and CV profiles in an electrochemical context using a simple machine learning (ML) algorithm, which continues to expand the integration of data science and electrochemistry.

## Introduction

Cyclic voltammetry (CV) is a conventional technique that has been used in the electrochemical context to justify the electrochemical behavior of any electrochemical system.^1^ This method serves as an effective tool for determining the electrochemical performance of energy storage, i.e., supercapacitor because it can provide detailed insights into the capacitive behavior, charge storage mechanisms, and electrochemical performance of supercapacitors.^2^ In fact, the CV responses are significantly affected by many experimental parameters, including electrolyte concentration, scan rates, electrode materials, potential window, and so on.^3^ These factors must be carefully optimized and controlled to ensure reliable and reproducible results.^1,4^ Moreover, the measurement of CVs typically requires substantial resources (e.g., time and chemical) in some extreme conditions such as superconcentration environment.^5^ Hence, this pain point can be addressed by using the machine learning (ML) algorithm to precisely predict those curves.^6^ Carbon-based materials are one of the best examples that can be used to demonstrate this novel application at early stages. This is because carbon materials have been widely used as EDLC electrodes due to their large specific surface area, excellent electrical conductivity, and remarkable chemical stability.^7,8^ Frackowiak and Béguin pointed out that activated carbon is one of the promising electrode materials for supercapacitors, owing to its commercial availability, low cost, and outstanding specific surface area (>2000 m^2^ g^–1^).^8^ Not only surface area but also porosity of the electrode materials plays a major role in the charge storage mechanism and ion diffusion capabilities of supercapacitors. The porous structure facilitates ion adsorption/desorption and formation of the electrical double layer at the electrode/electrolyte interface, resulting in improved specific capacitance and rate capability.^9^ These properties directly relate to the change of electrolyte concentration where the hydration size of the ions can be altered when changing the concentration.^5^ Hence, the electrolytes play a crucial role in determining the CV responses of the energy storage device, which affects the energy density (also refers to voltage and capacitance from the mathematical view). Typically, the low salt content is known to be aqueous electrolyte, while superconcentration (almost saturated) is known to be “water-in-salt” electrolyte.^10^ The “water-in-salt” electrolytes have attracted much interest, owing to their fast ionic transport, abundance, and high safety, attributed to their low toxicity and nonflammability.^10,11^ Yet, the voltage limit of the “water-in-salt” electrolyte was not restricted to 1.23 V due to the thermodynamics of water splitting.^11^ Suo et al. highlighted lithium bis(trifluoromethanesulfonyl)imide (LiTFSI) as a promising candidate for “water-in-salt” electrolytes, owning to its high solubility in water and robust hydrolysis resistance.^12^ Moreover, LiTFSI exhibits unique versatility, being soluble in both aqueous and nonaqueous electrolytes, and maintains chemical and electrochemical stability under both acidic and basic conditions.^13,14^ Therefore, machine learning (ML) has emerged as an efficient tool for CV prediction from large data sets to reduce the burden of actual measurement in vast scale, especially for the carbon-based electrode with water-in-salt electrolytes. This approach enables the rapid analysis and modeling of electrochemical systems, potentially streamlining experimental procedures in electrochemical research. Moreover, ML is a promising candidate for advancing scientific methodologies by reducing human error and maximizing the resource efficiency in experiments. Supervised learning is a critical technique utilized by computers to accurately predict outcomes by analyzing patterns in labeled input data. This method provides a robust basis for decision-making across various domains and enhances the precision of predictive models.^15^ This emphasizes the importance of updated techniques for evaluating experimental procedures to minimize resource consumption and boost efficiency in electrochemical research. Previous study demonstrated the use of artificial neural networks (ANN) for predicting the CV curve as an efficient and cost-effective technique. They proposed the software prototype so-called “Crypton 1.0” where the prediction were based on tuned ANN model with high prediction accuracy referred to the coefficient of determination (*R*^2^) of 0.98.^6^ However, one of ANN drawbacks is that it has been considered as “black box” models^16^ due to the lack of transparency in how the model process input data and derive their outputs, sometimes can results in untrustworthy prediction path which cannot be validated with real experiments, theories, and equations. Herein, the decision tree algorithm^17^ has been employed to extend the capability from the ANN model with expanded data set from our previous work to other type of activated carbon (YEC-8B). This method not only makes the prediction process more transparent but also facilitates easier validation and result interpretation.^18^ The decision tree regression is utilized to predict outcomes by considering the crucial input parameters such as concentration of LiTFSI, potential window, and scan rates. Additionally, data normalization and transformation were incorporated as preprocessing of data set to ensure the well-distributed data before training with ML algorithm. We adopted a 90:10 train–test ratio, enabling the model to learn from most of the data while preserving a sufficient portion for validation. The use of decision tree regression involved the utilization of continuous data in order to provide predictions at the leaf nodes of the tree.^19,20^ This method ensures a robust framework for accurate and interpretable results in the analysis of electrochemical systems.

## Methodology

### Chemical and Methodology

The three-electrode system was employed to study CV of the activated carbon from aqueous ("salt-in-water") to “water-in-salt” electrolyte using LiTFSI as a based salt. YEC-8B (Fuzhou Yihuan Carbon, China) activated carbon with surface area of about 2000 m^2^ g^–1^ was used as an electrode active material. The double junction Ag/AgCl and polycrystalline platinum wire were served as reference and counter electrodes, respectively. To prepare the working electrode, YEC-8B was combined with carbon black and poly(vinylidene fluoride) in a weight ratio of 8:1:1. This mixture was then dispersed in the NMP solvent in a weight ratio of 1:9, to obtain an electrode slurry. Subsequently, 2 μL of the slurry was dropped on a polished glassy carbon with a mass loading of 2.83 mg cm^–2^ and dried at 60 °C for 12 h prior to electrochemical measurement. To study insights into the behavior of both “salt-in-water” and “water-in-salt” regimes, the electrolyte was prepared at different concentrations of 1, 5, 10, and 20 *m*, respectively. Note that the conductivity values for these concentrations were measured as 29.2, 47.0, 32.6, and 12.7 mS cm^–1^, respectively. The viscosity values for the same concentrations were measured as 1.25, 2.59, 5.47, and 19.99 mm^2^ s^–1^ (see Figure S1). Note that electrolyte properties are reported in previous literature.^21^

### Cyclic Voltammetry Data Collection and Interpretation

**Figure 1:**
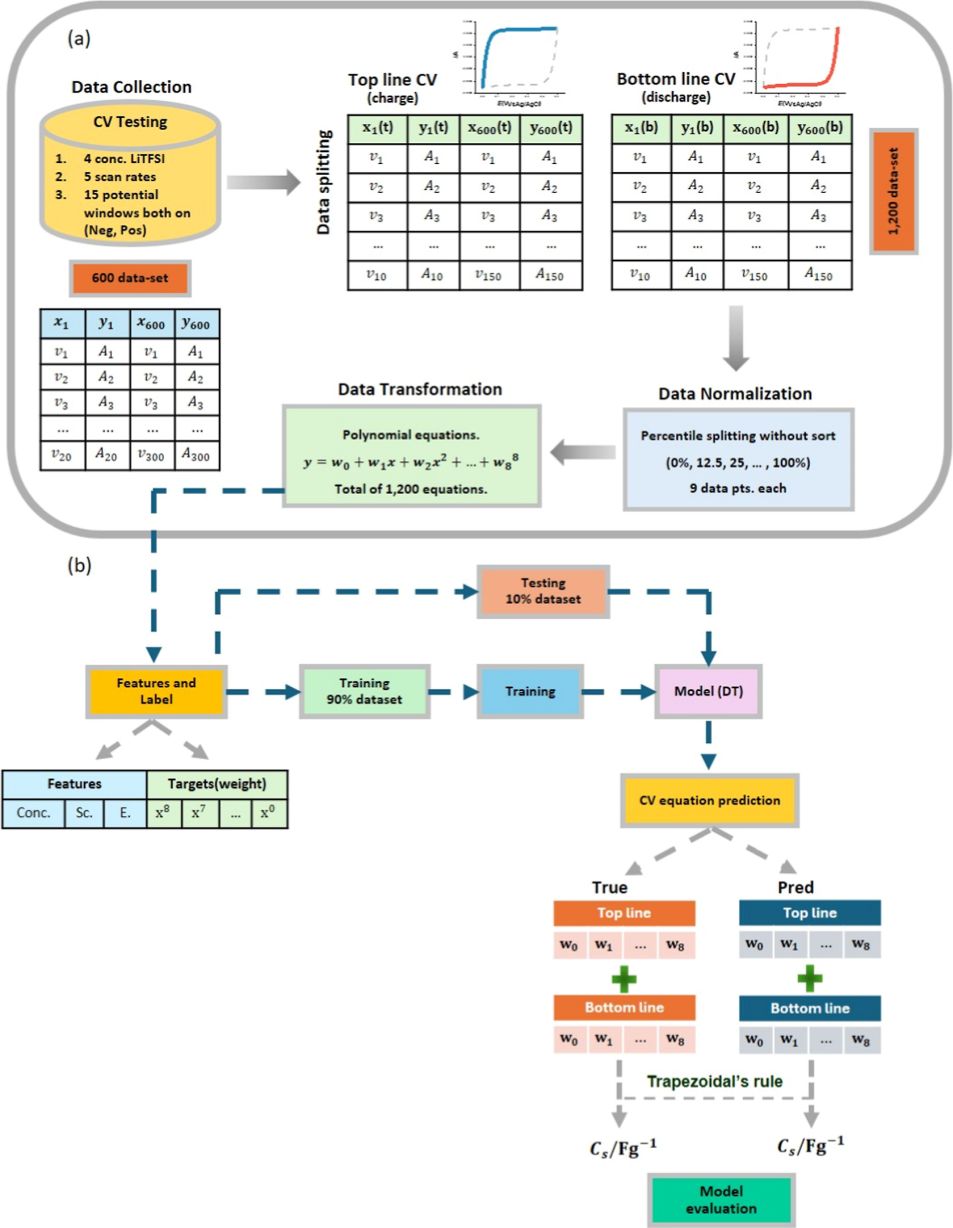
Schematic showing the data preprocessing and ML design workflow for voltammetry prediction.

The CVs were collected using PalmSense4 running PSTrace5 software. The CV measurement was carried out in house at different scan rates 10, 25, 50, 75, and 100 mV s^–1^ (the data were collected by performing three replicates of each scan rate), and it was swept between 0 and ±1.5 V vs Ag/AgCl on both wings. Note that comparative study with YEC-8A was outlined in our previous report.^6^ The data were then collected by manipulating three key features that contribute to the energy storage performance, which are (i) concentration of electrolytes-from “salt-in-water” to “water-in-salt”, (ii) charge/discharge rate, referring to scan rates, and (iii) operating voltage. In total, the as-collected data were 600 data sets, as shown in Figure 1a. The CVs were recorded with a step potential of 0.1 V and kept expanded from 0.1 to 1.5 V. For the step potential of 0.1 V, it contains 20 data points. Thus, the potential window of 1.5 V existed for 300 data points.

The charge storage process observed in CV can be distinguished into two processes, charging and discharging. The charging profile is represented by the top line (forward scan), which begins at a low potential and progresses toward a high potential. Conversely, the discharge profile is depicted by the bottom line (reverse scan), starting at a high potential and moving toward a lower potential.^1^ The reason for separating CV profiles into “top line” and “bottom line” is because the different charge storage processes provide different electrochemical characteristics. Therefore, the data for each cycle consisted of 1200 data sets, with 600 for charging and 600 for discharging.

However, the unequal distribution of data points was found in our data set. To address this issue, a normalization method using percentile splitting has been implemented. This technique divides the data into percentiles without sorting, starting at the zeroth percentile with a minimum data set size of 10 data points and extending to the 100th percentile. The data were split into intervals of 12.5%, ensuring each subset contains nine data points. According to the shape of the CV, polynomial fitting was employed for data transformation. The optimal fit was achieved by selecting a polynomial degree of “*n* – 1”, where *n* represents the number of data points.^22^ Thus, for a data set containing nine data points, an eighth degree (octic) polynomial is applied. These octic polynomial equations are then incorporated into our ML model to enhance prediction accuracy.

### Machine Learning Models

The key feature that plays a significant role in the CV shape for carbonaceous materials is reported to be the type of activated carbon, electrolyte, scan rate, and operating voltage. Those parameters were defined as “conc.”-concentration of LiTFSI, “*E*”-potential window, and “Sc”-scan rates, as shown in Figure 1b. The CV of commercial YEC-8B were reported in our previous work.^6^ The result is a weight of polynomial ranging from *y* = *x*^8^ + *x*^7^ + *x*^6^ + *x*^5^ + *x*^4^ + *x*^3^ + *x*^2^ + *x*^1^ + *x*^0^. A total of nine targets were gathered in a CSV file, which was available to download via Supporting Information. Hence, the data were split into 90% for the training set and 10% for the test set. Given the wide range of data produced by the octic polynomial equation, we applied min–max scaling to normalize the data within the 0 to 1 range. This technique simplifies and structures the data, facilitating better visualization and easier model evaluation.^23,24^ The Decision Tree model in ML is versatile, handling both supervised and unsupervised learning tasks. It adapts flexibly to diverse data sets and delivers rapid predictions, making it highly suitable for applications in electrochemical problem-solving.^19,25−27^ Furthermore, this study involves nine continuous target variables, allowing for the formulation of clear and interpretable rules for prediction.^26^ This clarity and ease of understanding are why the decision tree regression model was employed in this work. This model has the capability of providing 540 data sets for both the top half of the CV model and the bottom half of the CV model in the train set. In addition, 60 data sets were selected as the test set, and the random state was set to 0 of both top halves and bottom half model. The model was optimized by using a minimum sample split of 2 and a minimum sample leaf of 1. The mean absolute percentage error (MAPE) was used as an evaluation metric. This variable can quantify the average percentage difference between the predicted values and measured values (value from the experiment). Lastly, it is possible to use the trapezoid’s rule to integrate the area of the CV curve which directly provides the capacitance value. It is shown that the method can give good accuracy of integration.^28^ Following the collection of the area required to compute capacitance using the integral product of the area, the area was obtained using the numerical technique followed eq 1
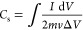
1where *m* is the mass loading of the sample, *v* represents the scan rate, *I* d*V* is the integral area under the CVs, and Δ*V* is the operating voltage window of the CVs.

## Results and Discussion

### Electrochemistry of YEC-8B in LiTFSI

**Figure 2:**
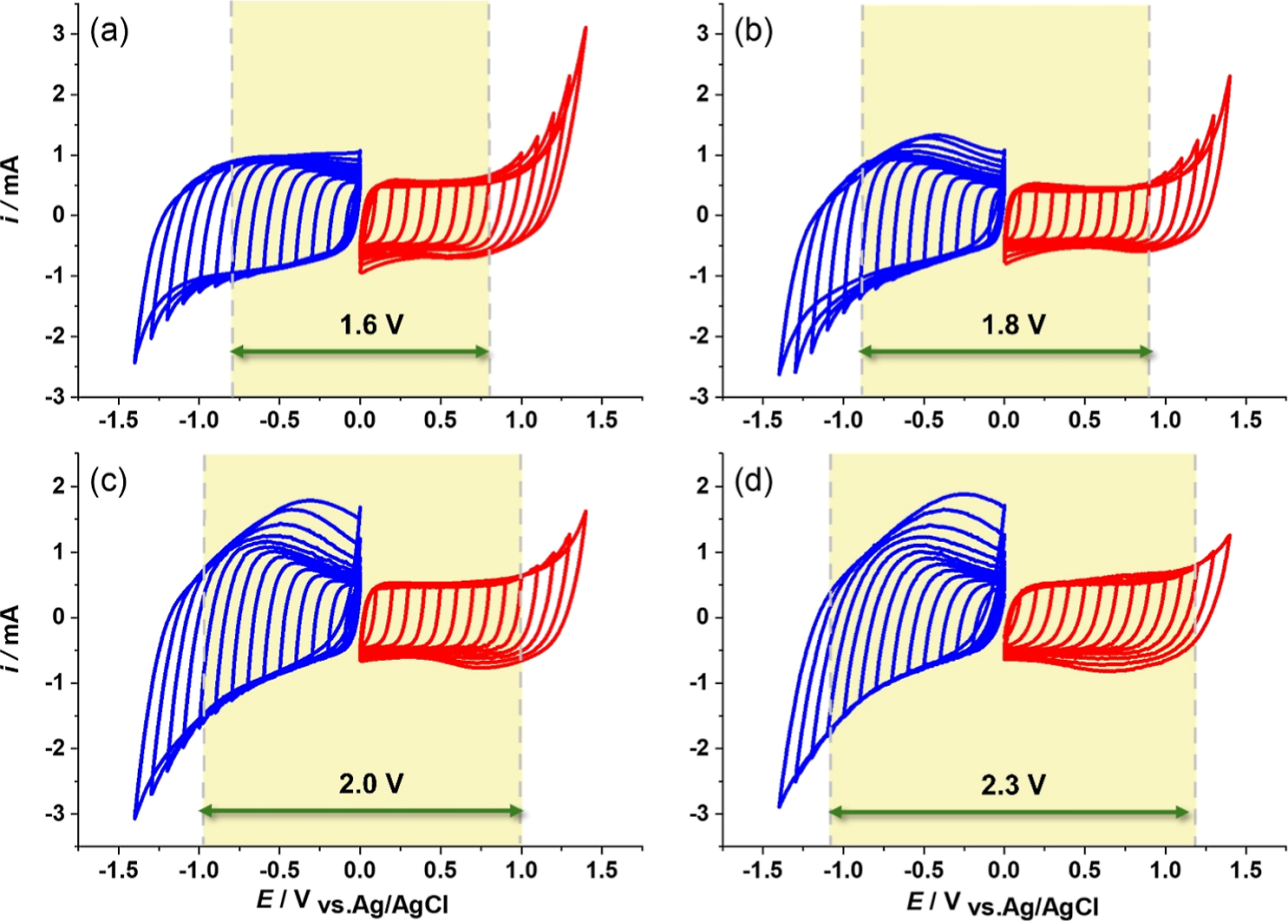
CV profiles of YEC-8B at 10 mV s^–1^ in a variety of LiTFSI concentrations: (a) 1, (b) 5, (c) 10, and (d) 20 *m*. The red curves represent the positive sweeps while the blue curve represents negative sweeps.

A large electrochemical stability window (ESW) is required for improving energy density of carbon-based supercapacitors.^29,30^ However, in aqueous electrolytes, cycling the electrode to extremely high potentials can lead to electrolyte decomposition and the evolution of gases such as hydrogen and oxygen.^31^ Previous studies have demonstrated that both electrolyte concentration and electrode material significantly influence the capability for gas evolution in electrochemical systems.^6,32−35^ Therefore, optimizing the operating potential window is essential to prevent gas evolution, which can degrade the performance and lifespan of electrode materials. However, this optimization could affect the shape of the CVs in the data collection processes. The electrochemistry of YEC-8B was carried out in a variety of electrolyte concentrations from “salt-in-water” (refer to 1 *m* LiTFSI) to “water-in-salt” (20 *m* LiTFSI), as shown in Figure 2. The tails observed at the positive and negative end of the CV profile indicate the oxygen and hydrogen evolution reaction, respectively.^12^ This positive and the negative limit of the CV response was expanded when applying a higher salt concentration. The results from CV measurements confirm that increasing the concentration of the electrolyte can effectively expand the potential window, thereby preventing gas evolution, particularly oxygen evolution during charging. For a low concentration (1 *m*), the CVs response in Figure 2a show a quasi-rectangular-like shape at the yellow regime (from −0.8 to 0.8 V vs Ag/AgCl), indicating ideal capacitive behavior of the carbon-based materials. However, the CV shapes for positive and negative sides are slightly different due to the preference of cations/anions adsorption on the carbon surface.^36^

The CV on the negative branch shows higher adsorbed charges (integrated area under CVs) compared to the positive branch, referring to the higher capacitance (Figure S2). At a concentration of 5 *m* LiTFSI (Figure 2b), the voltage window can be expanded to 1.8 V (from −0.9 to 0.9 V vs Ag/AgCl). The hump peak is present at ca. −0.7 to −0.2 V vs Ag/AgCl, indicating the insertion of desolvated ions into the ultramicropore of the activated carbon.^37^ These hump peaks were shifted to lower voltage when applied to higher LiTFSI concentration, where the 5, 10, and 20 *m* LiTFSI provide the peak at about −0.50, −0.35, and −0.25 V vs Ag/AgCl, respectively (Figure 2c,d). Notably, the 20 *m* LiTFSI exhibits the largest operating potential window, extending up to 1.2 V on the positive side and −1.1 V on the negative side. This can be employed to obtain the electrochemical stability windows for electrolytes at concentrations of 1, 5, 10, and 20 M as 1.6, 1.8, 2.0, and 2.3 V, respectively.

### Capacitive Behavior of YEC-8B in LITFSI

**Figure 3:**
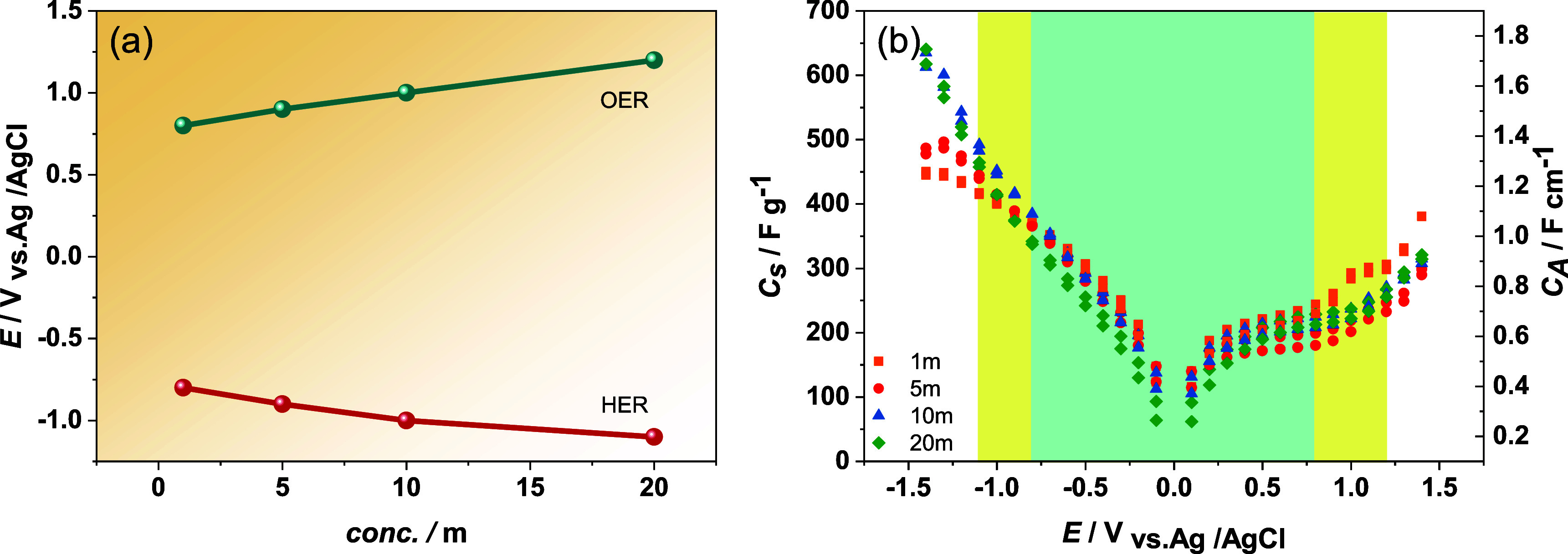
(a) HER and OER reactions in 1, 5, 10, and 20 *m* LiTFSI, depicted with the potential window on the *y*-axis. (b) Capacitance measurement, with the left *y*-axis presenting specific capacitance (per active mass loading), and the right *y*-axis presenting areal capacitance (per area of the electrode).

Regarding the variation in the potential range during CV in Figure 2, the positions of the OER and HER peak at different electrolyte concentrations are plotted in Figure 3a. The results show that higher electrolyte concentrations offer a wider electrochemical window, as confirmed by the shift of the OER and HER reactions toward higher overpotentials. Especially, the OER peak shifts from 0.8 to 1.2 V, when increasing the LiTFSI concentration from 1 to 20 *m*, while the HER peak shifts from −0.8 to −1.1 V, indicating an increased overpotential. Therefore, it is suggested that the OER is mainly contributed by the concentration of the electrolyte.^38^ The increased overpotential refers to the more potential required to drive the OER and HER reactions. This suggests that higher electrolyte concentrations enhance stability and resistance to decomposition due to a reduced number of free water molecules.^39,40^ The specific capacitance and areal capacitance can be calculated from CV curves at the same scan rate of 10 mV s^–1^ but at different potentials, as shown in Figure 3b. As the operating potential window is increased, the capacitance is directly enhanced, owing to the correlation between capacitance and charge storage within the applied potential range. A wider potential window allows for more charges to be stored as the system can accommodate more charge carriers within the expanded potential range, resulting in higher capacitance. However, for electrolytes with lower concentrations (1 and 5 *m* LiTFSI), the capacitance exhibits plateau limits at high potentials (yellow zone), attributed to the charge storage limitations caused by electrolyte decomposition or water splitting. In the case of 1 *m* LiTFSI, high capacitance can be observed at high potentials, but this is from unwanted reactions of electrolyte decomposition at high potentials which can lead to poor cycling stability of supercapacitors.^41^ In contrast, electrolytes with higher concentrations of LiTFSI (10 and 20 *m*) show a nearly linear relationship between capacitance and applied voltage, suggesting that higher concentration electrolytes can maintain their charge storage capabilities over a wider potential range, leading to higher capacitance and wider stable window potential.^42^

To study the kinetic properties of the LiTFSI electrolyte at different concentrations on electrochemical behavior, CV measurements of the YEC-8B electrode were performed at different scan rates from 10 to 100 mV s^–1^ (Figures S2–S5). *E*^+^ represents the calculated capacitance from the negative sweep, while *E*^–^ represents the capacitance from the positive sweep. The specific capacitance from positive sweep (*E*^+^) shows nearly consistent values and a similar rate capability across different electrolyte concentrations. However, the impact of the electrolyte concentration on the negative sweep (*E*^–^) is more pronounced. This difference can be attributed to the distinct diffusion dynamics of the Li^+^ cation and the TFSI^–^ anion. The Li^+^ cation is smaller and lighter, which may result in significant diffusion rate changes under varying scan rates during negative sweep.^43^ Although 20 *m* shows the highest specific capacitance (507.66 F g^–1^) at 10 mV s^–1^ due to superior charge storage capability, 5 *m* LiTFSI exhibits the highest rate capability with capacitance retention of 64.13% after increasing scan rate up to 100 mV s^–1^. This superior rate capability is attributed to the high electrical conductivity of the 5 *m* LiTFSI electrolyte (see Figure S1d), which facilitates efficient ion transport and minimizes resistance during cycling at a high rate.^44^ Therefore, not only specific capacitance but also operating window potential and electrical conductivity of the electrolyte need to be optimized to achieve high-performance supercapacitors with long-term cycling stability.

### Prediction Accuracy and Model Performance

**Figure 4:**
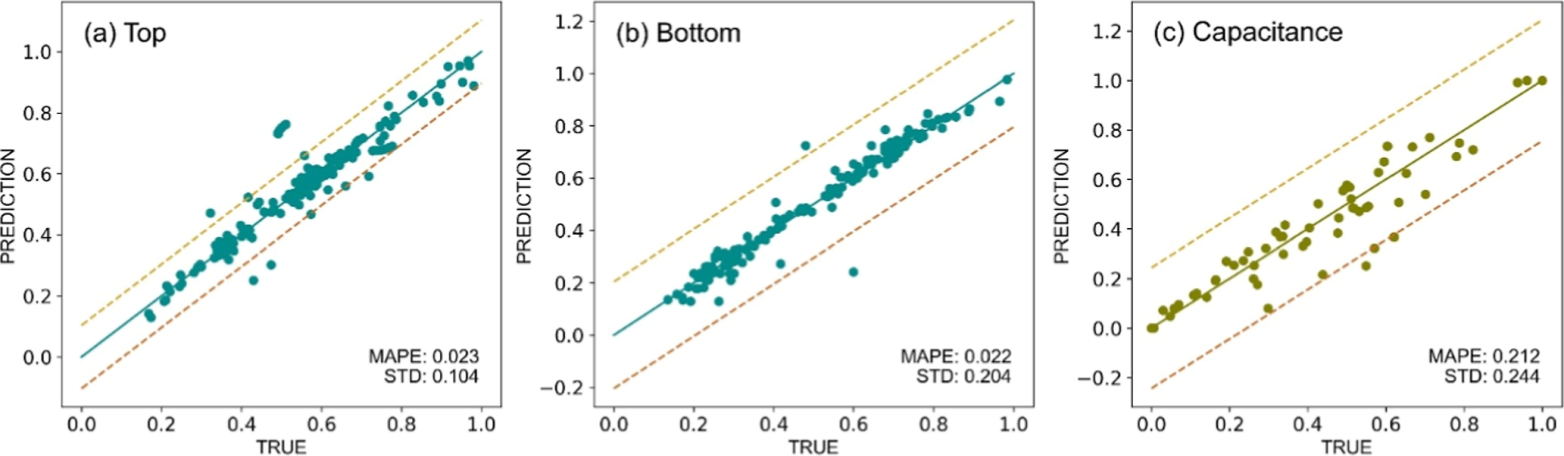
Scattered plot of the experimental and predicted value. (a) Top half CV model, (b) bottom half CV model, and (c) calculated capacitance model. The dash yellow line represents +1 standard deviation margin while the red dash line represents −1 standard deviation margin from the reference diagonal line. The label of the MAPE and standard deviation values were provided at the right bottom corner of each subplot.

The performance of the model is summarized in Figure 4 using scatter plots that illustrate the comparison between the predicted values on the *y*-axis and the experimental values on the *x*-axis. Figure 4a shows that the top half CV model has a standard deviation of 0.104, whereas Figure 4b displays the bottom half CV model with a comparative standard deviation of 0.204. According to the similar standard deviation between the top half and bottom half of the CV model, this indicates the consistency of prediction accuracy for the complete CV profile. Consequently, the decision tree techniques could be used to create models that reliably forecast the weights of nine outputs that correspond to each polynomial profile of input distribution. In order to further evaluate the predictive ability of the developed model, the unseen test was performed with 60 data sets which are not included in the initial training procedure. The MAPE is used as the main indicator for determining the model accuracy among other indicators as combined in Table S1 (see the Supporting Information for more detail). As a result, the MAPE for the top half CV model is 0.023, which is considered as a small error of only 2.30%. Similarly, the bottom half of the CV model has MAPE of 0.022, which is equivalent to a small error of 2.20% as well. Regarding Figure 4c, the model specifically provides corresponding capacitance values from calculation based on predicted CV profile, achieving MAPE of 0.212 which is substantially higher than the MAPE of predicted CV itself. To be precise, this indicates that the capacitance values have an error margin of 21.20% with a standard deviation of 0.244. Therefore, this confirms that the capacitance calculation based on the predicted CV profile tends to hold more deviation than the prediction of the CV profile around 10%. In addition, the feature importance analysis has been performed; the concentrations of LiTFSI, potential window, and scan rate are identified as significant factors. For the top half of the CV model, their respective unitless important values are 0.087, 0.777, and 0.136. For the bottom half CV model, the importance values of those features are 0.155, 0.682, and 0.163, respectively. According to the degree of importance, when it comes to estimating the weight of a polynomial as the objective variable, the potential window is considered as the key feature in determining the shape of the CV curves with the highest degree of importance scoring. Thus, this can shed some light on the importance of the potential window over other features in the modeling process. In addition, the dominant effect of potential windows on the determination of CV curve behavior is well-correlated with the direct measurement from the actual experiment as well.

**Figure 5:**
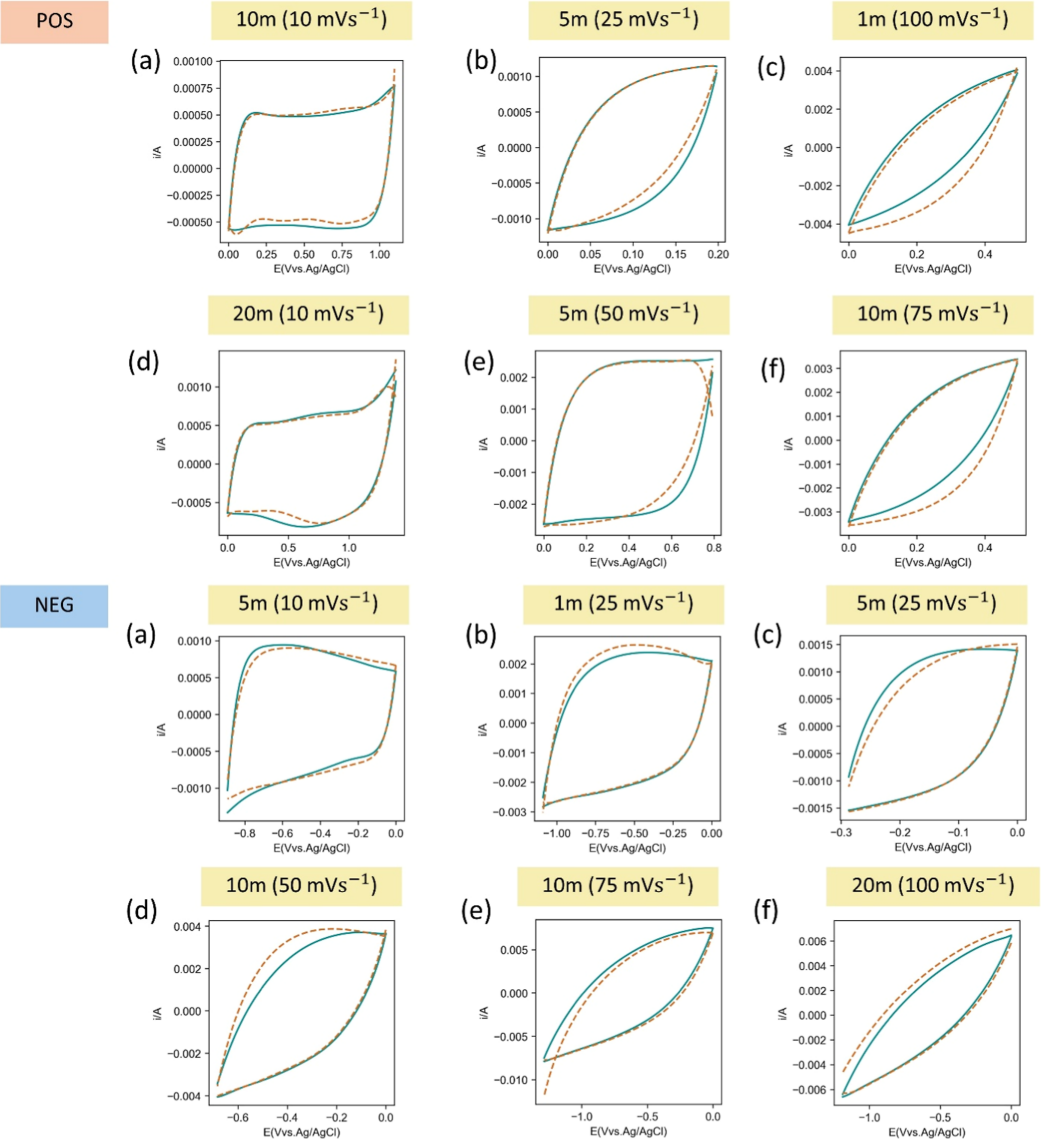
Negative (NEG) and positive (POS) half scans of CV prediction per LiTFSI concentration and scan rate. Please note that the orange dash line represents the prediction curve, and green solid line represents the experiment profile.

To clarify the different behavior of CVs and the corresponding prediction accuracy, the different comparative CV profiles (experimental versus predicted curves) at selected LiTFSI concentrations and scan rates within unseen data set are combined in Figure 5. The complete illustration of comparative CV profiles with systematic variation of LiTFSI concentrations and scan rates is combined in Supporting Information (see Figures S6–S13). Figure 5a represents profiles with the negative potential window (negative half profiles), and Figure 5b exhibits the profiles with the positive potential window (positive half profile). After a thorough investigation, it is very clear that our proposed decision tree model can provide accurate CV prediction of 9 profiles out of 12 profiles which is approximately 75% accuracy. Consequently, the adequate boundaries of the potential windows for accurate prediction can be determined by investigation of respective predicted profiles. Ensuring that the coefficient of the polynomial equation, so-called eighth-degree polynomial, is determined based on these boundary ranges inside the test set, it is very clear that accurate prediction dominantly occurred for wide potential windows which exceed 0.5 V up to 1.0 V and a scan rate of 10 mV s^–1^ at different electrolyte concentrations. While slower scan rates tend to provide more deviated predicted CV profiles which directly deteriorate their predictive accuracy. Thus, the obtained boundaries of potential window and scan rate demonstrate a well-correlation to the experimental control variables and the previous summary from our previous work with usage of ANN.

**Figure 6:**
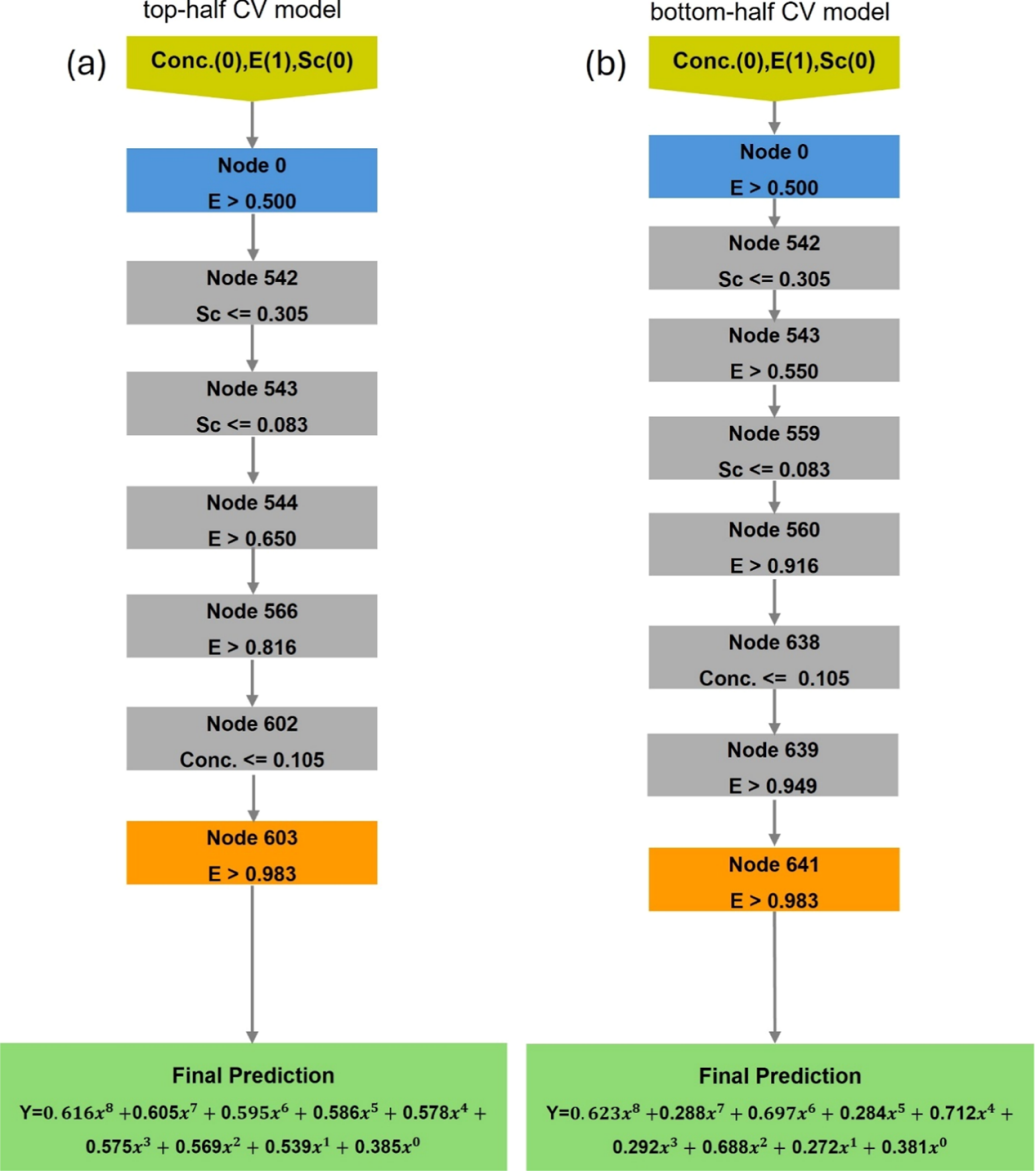
Specific artificial data points and examining the decision rules of the top half CV model and bottom half CV model by ranging from 0 to 1 of LiTFSI concentration (“conc”), potential window (“*E*”), and scan rate (“Sc”).

The final evaluation of our proposed decision tree model is performed to additionally ensure its reliability apart from the unseen test. The thorough analysis is conducted based on decision rules by using artificial input data to forecast additional outputs and generate extended decision tree branches. This operation tends to provide the extensive prediction ability of the original decision tree model through multiple artificial values corresponding to the respective input features, which are LiTFSI concentration, scan rates, and potential window. Please note that the artificial input data are standardized on a scale from 0 to 1. According to the feature importance score, the potential window tends to provide a dominant effect on determining the behavior of CV profiles. Therefore, the decision rules were set accordingly by setting potential window (“*E*”) to 1, while both concentration of LiTFSI (“conc”) and potential window (“*E*”) are set to 0, as illustrated in Figure 6. This extensive prediction process involves both the top half and bottom half models, which ultimately leads to the construction of a complete CV. The decision rule initiates at root node 0 (initial node), traversing decision nodes with specific rules denoted^26,45^ labeled as (a) and (b) which correspond to the top half and bottom half model, respectively, as shown in Figure 6. Afterward, the stop criteria are entertained once the value of potential window approaches 1 which results in the node finalization at 603 for the top half CV model and 641 for the bottom half CV model. Finally, the eighth polynomial equation prediction is generated to complete the reference equation corresponding to the full CV profile which can be further used to determine the capacitance value. Hence, this extensive analysis can successfully provide the reference equation for generating CV profile within standardized range of respective input features, which generally refers to the boundary of accurate prediction from the unseen test.

**Figure 7:**
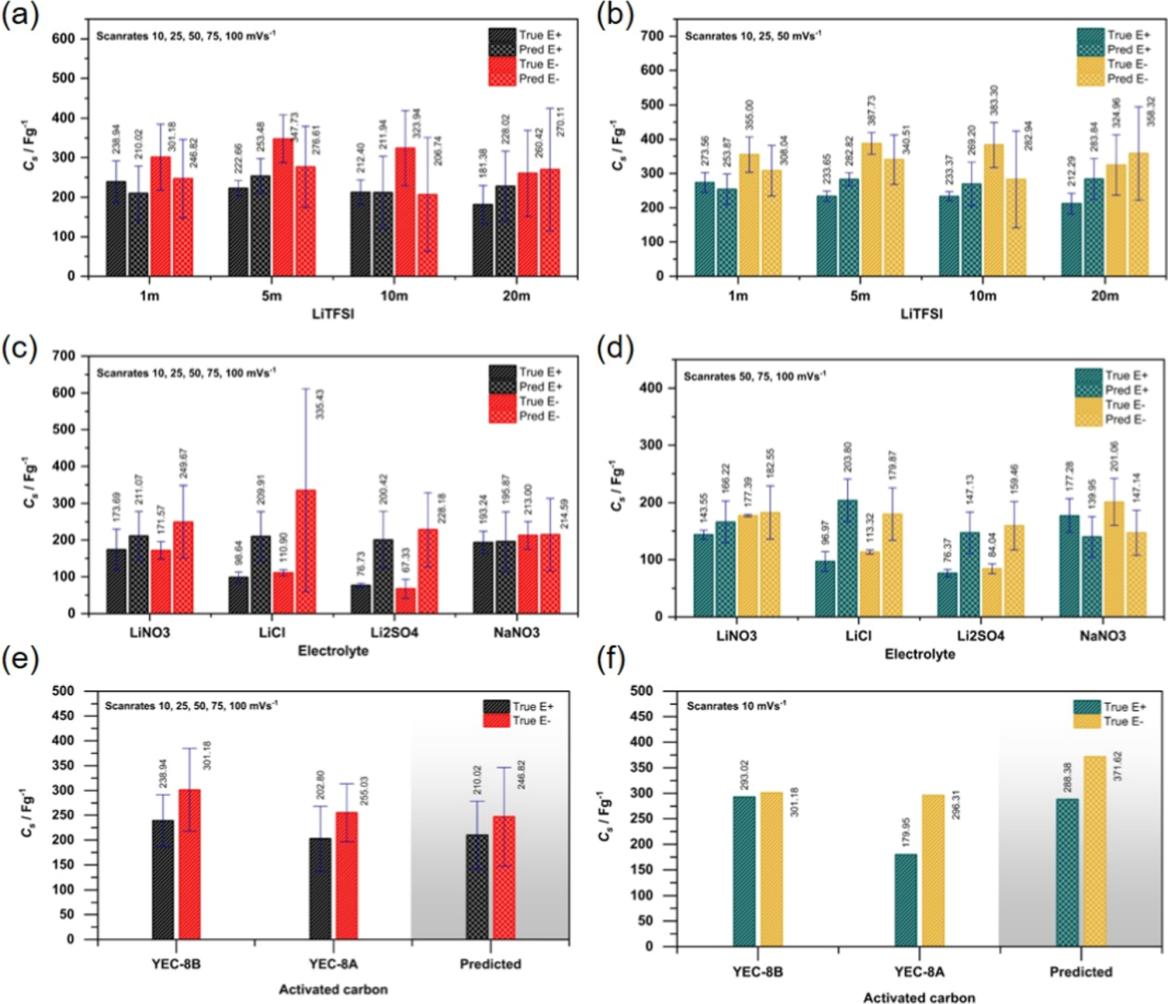
Comparison of capacitance obtained from the predictions and the as-measured (true values) for specific scenario. Left plots represent the result from all scan rates from 10 to 100 mV s^–1^, while the right plots show the capacitance at low scan rates from 10 to 50 mV s^–1^. (a,b) Effect of concentrations of LiTFSI, (c,d) effect of electrolytes, and (e,f) effect of activated carbon structural.

To evaluate the model ability for capacitance prediction on actual data (also refer to unseen new data), the predictions were validated with four concentrations of LiTFSI (1, 5, 10, and 20 *m*) using YEC-8B activated carbon. Predictions were made for both the negative (represented by black bars) and positive potential windows (represented by red bars), as illustrated in Figure 7a. To analyze the effect of concentration on predictions, the potential window was fixed at ±1.0 V vs Ag/AgCl for both the negative and positive windows, while the scan rate was varied across five values (10, 25, 50, 75, and 100 mV s^–1^). The results demonstrated a strong agreement between predicted and experimental capacitance values. For instance, at a concentration of 1 *m* in the positive potential window, the experimental capacitance was 238.94 F g^–1^, while the model predicted 210.02 F g^–1^, corresponding to an error of approximately 12% with almost equivalent of standard deviation. To minimize the error and optimize prediction accuracy, a suitable range of scan rates was identified, as shown in Figure 7b. By reducing the scan rates to three values (10, 25, and 50 mV s^–1^) the prediction error decreased to approximately 7%. This optimized range of scan rates significantly improves the reliability of the predictions by reducing the overall error. It should be noted that prediction at low scan rates provides more reliable results than that of high scan rates.

For effect of salts (electrolyte types), the validation data set was prepared using a fixed concentration of 1 *m* for four salts (LiNO_3_, LiCl, Li_2_SO_4_, and NaNO_3_) on YEC-8B activated carbon. To further test the model, predictions were conducted across varying potential windows specific to each salt. This variation aligns with the importance of high ionic conductivity and wide electrochemical stability windows for supercapacitor applications.^46^ For LiNO_3_, the potential range for a full CV cycle was 2.18 V, while for LiCl and Li_2_SO_4_, the ranges were 2.06 and 1.33 V, respectively.^21^ For experimental consistency, the potential windows for the negative and positive scans were set to 1.09 V (LiNO_3_), 1.03 V (LiCl), and 0.66 V (Li_2_SO_4_). Additionally, NaNO_3_ was included as a representative nonlithium salt for model trial,^47^ with a selected potential window of 0.55 V applied for both positive and negative CV scans. Figure 7c illustrates the prediction results for various salts. For example, the average prediction error for LiNO_3_ across both potential windows was approximately 33.5%. However, by optimizing the scan rate range to 50, 75, and 100 mV s^–1^ on Figure 7d, the error was reduced to 9.35%. For the other salts, the model exhibited prediction errors of 84.4% for LiCl, 91.2% for Li_2_SO_4_, and 23.93% for NaNO_3_. In summary, the model demonstrated the capability to predict the capacitance across various salts with varying degrees of accuracy. When ranking the salts based on the prediction performance, the percent error followed the order: LiNO_3_ > NaNO_3_ > LiCl > Li_2_SO_4_, under a fixed 1 *m* concentration and differing potential windows. This analysis highlights the model’s potential for salt-specific capacitance predictions.

The model predictions are influenced not only by the concentration of electrolytes (1 M in this case) but also by the materials used. The validation data set, derived from 1 *m* LiTFSI electrolyte and YEC-8A activated carbon, was used to predict the potential window at 1 V, considering both positive and negative potential windows across five different scan rates. A comparative study with KOH electrolyte showed that YEC-8B exhibited a higher specific capacitance in the KOH electrolyte.^48^ This finding was confirmed by the validation data set for LiTFSI, where YEC-8B demonstrated a higher specific capacitance than YEC-8A at ±1.0 V vs Ag/AgCl in both the negative and positive potential windows, as shown in Figure 7e. The model predictions closely align with the capacitance values for YEC-8A as their physical properties are similar.^48^ The optimized scan rate range for accurate predictions was determined to be 10 mV s^–1^ as shown in Figure 7f. Additionally, the model predicted a 43% shift in the suitable scan rate range for YEC-8A activated carbon in 1 *m* LiTFSI across both potential windows.

## Conclusions

To summarize, the proposed decision tree model can accurately predict the correlation between the CV curve and the significant input parameters with an approximate accuracy of 75%. The size of the potential window has a direct impact on the CV curve, with larger windows resulting in larger curves. Through the utilization of this model, we can investigate the ESW of LiTFSI electrolytes with YEC-8B activated carbon, which has the potential to reach a maximum of 2.3 V by utilizing a 20 *m* aqueous LiTFSI electrolyte. In addition, the decision tree model has the capability to forecast up to 9 targets out of 12 targets from unseen data set with polynomial curve features and examine the weight of both the upper and lower lines of the CV curve. The potential window boundary and the suitable scan rate are proposed to be within range of 0.5 to 1.0 V and 10 mV s^–1^, respectively, which well-correlated to the nature of actual measurement under electrochemical context and our previous findings with ANN. Consequently, a simple tree-based regression algorithm can provide comparable prediction ability to the more complex ML algorithm which tends to eventually reduce the computation burden for future work.

## Supporting Information Available

Additional experimental and results sections; experimental section encompassing the utilization of various chemicals, materials, and the measuring of electrolytes; and extent of ML prediction findings (PDF)
