# Supplementary material for: Voltammetry Prediction and Electrochemical Analysis of Carbon Material from “Salt-In-Water” to “Water-In-Salt”
Source: Anal Chem. 2025 Jan 31;97(7):3881–91. doi: 10.1021/acs.analchem.4c04764 (PMC11866287; doi:10.1021/acs.analchem.4c04764)
Supplement: Supplementary file 1 — ac4c04764_si_001.pdf [file ac4c04764_si_001.pdf]

Supporting Information

Voltammetry Prediction and Electrochemical Analysis of Carbon Material from "Salt-in-water" to "Water-in-salt"

Sukanlaya Kornnum[a,b], *Praeploy Chomkhuntod*[a,b], *Nick Schwaiger*[a], *Kanwara Limcharoen*[a], *Krittapong Deshsornand*[a,b], *Kulpavee Jitapunkul*[c], *Pawin Iamprasertkun*[a,b,*]

[a]*School of Bio-Chemical Engineering and Technology, Sirindhorn International Institute of Technology, Thammasat University, Pathum Thani, Thailand 12120 (pawin@siit.tu.ac.th)*

[b]*Research Unit in Sustainable Electrochemical Intelligent, Thammasat University, Pathum Thani, Thailand 12120*

[c]*Department of Chemical Engineering, Faculty of Engineering, Kasetsart University, Bangkok, Thailand 10900*

Corresponding Author

*P. Iamprasertkun (email: pawin@siit.tu.ac.th) Tel: +66-2-986-9009 ext. 2306

https://orcid.org/0000-0001-8950-3330,

Keywords: Energy storage, Machine Learning, Decision tree, Cyclic Voltammetry, Activated Carbon

Table of Content

Content	Page
Electrolyte properties of LiTFSI	S1
Electrochemical properties in each electrolyte concentrations	S3-S5
Electrochemical properties in YEC-8A activated carbon	S6
Electrochemical properties in various salts	S6-S8
Metric of evaluation	S9
Example of CV prediction on negative branch	S10-S13
Example of CV prediction on positive branch	S14-S17
GitHub repository	S18

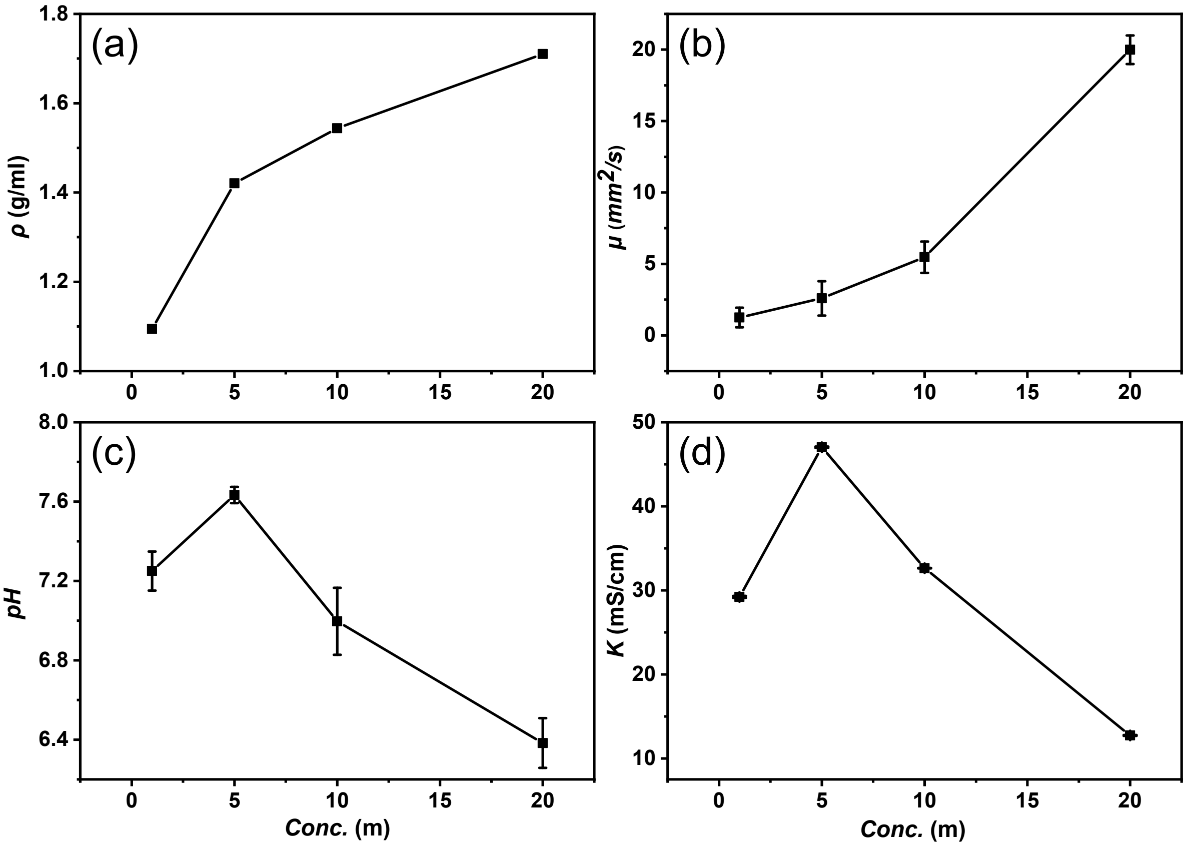

Figure S1: The properties of LiTFSI electrolyte from aqueous to "water-in-salt" concentration. (a) density, (b) viscosity, (c) pH, and (d) electrical conductivity.

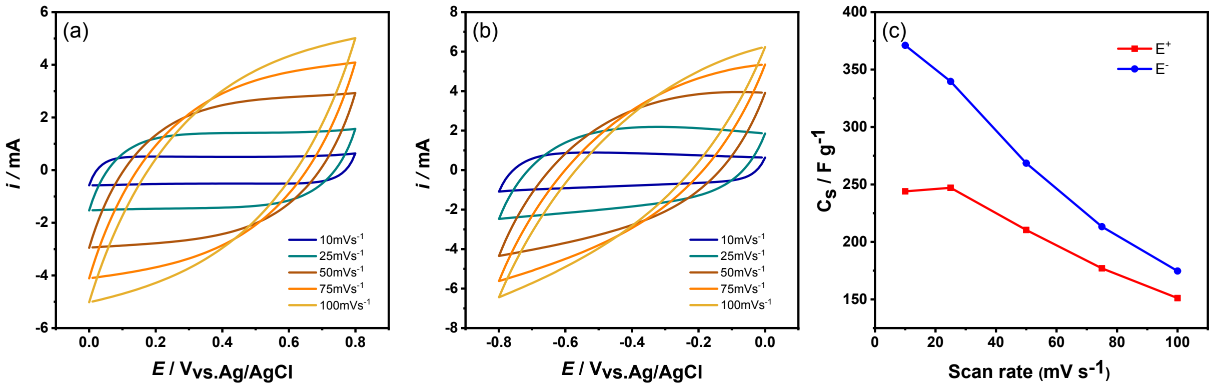

Figure S2: Electrochemical properties of 1 m LiTFSI, CVs from 10 mV s^{-1} to 100 mV s^{-1} (a) positive scan, (b) negative scan, and (c) specific capacitance vs. scan rates.

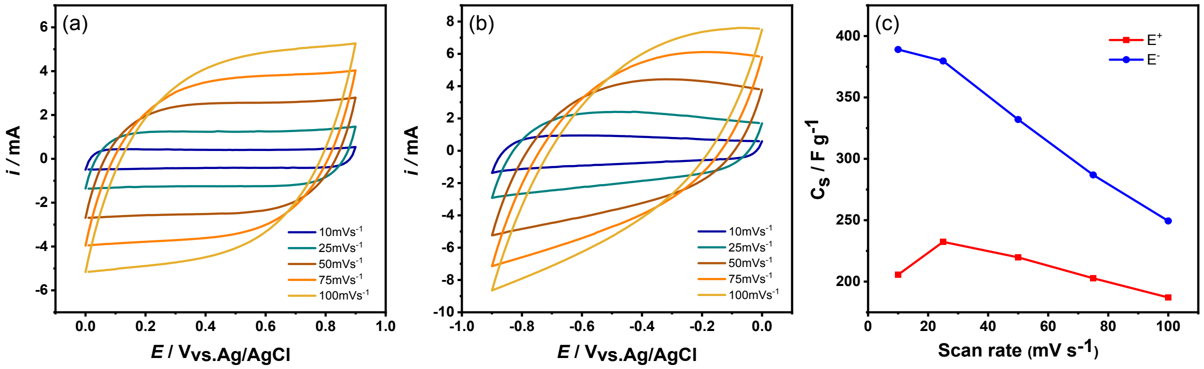

Figure S3: Electrochemical properties of 5 m LiTFSI, CVs from 10 mV s^{-1} to 100 mV s^{-1} (a) positive scan, (b) negative scan, and (c) specific capacitance vs. scan rates.

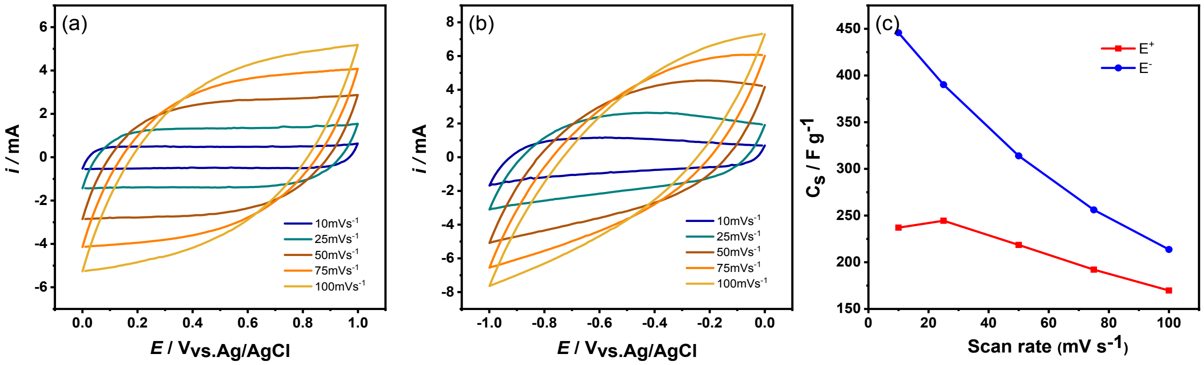

Figure S4: Electrochemical properties of 10 m LiTFSI, CVs from 10 mV s^{-1} to 100 mV s^{-1} (a) positive scan, (b) negative scan, and (c) specific capacitance vs. scan rates.

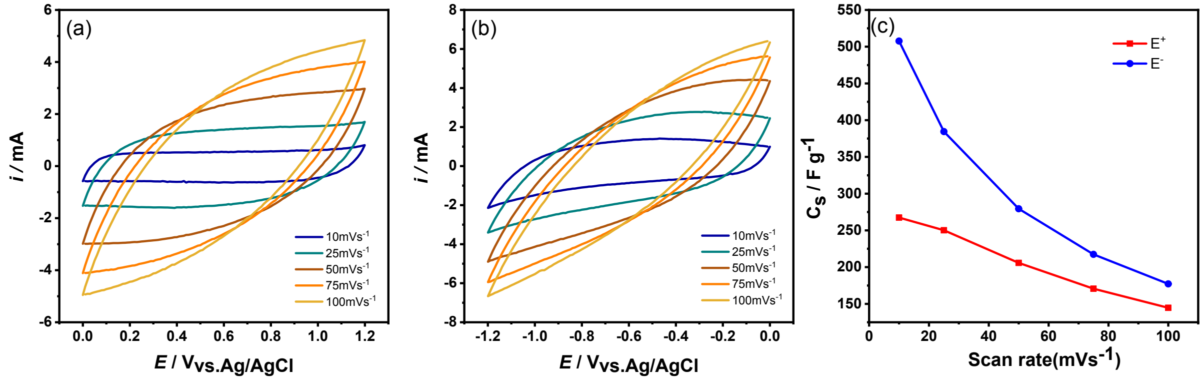

Figure S5: Electrochemical properties of 20 m LiTFSI, CVs from 10 mV s^{-1} to 100 mV s^{-1} (a) positive scan, (b) negative scan, and (c) specific capacitance vs. scan rates.

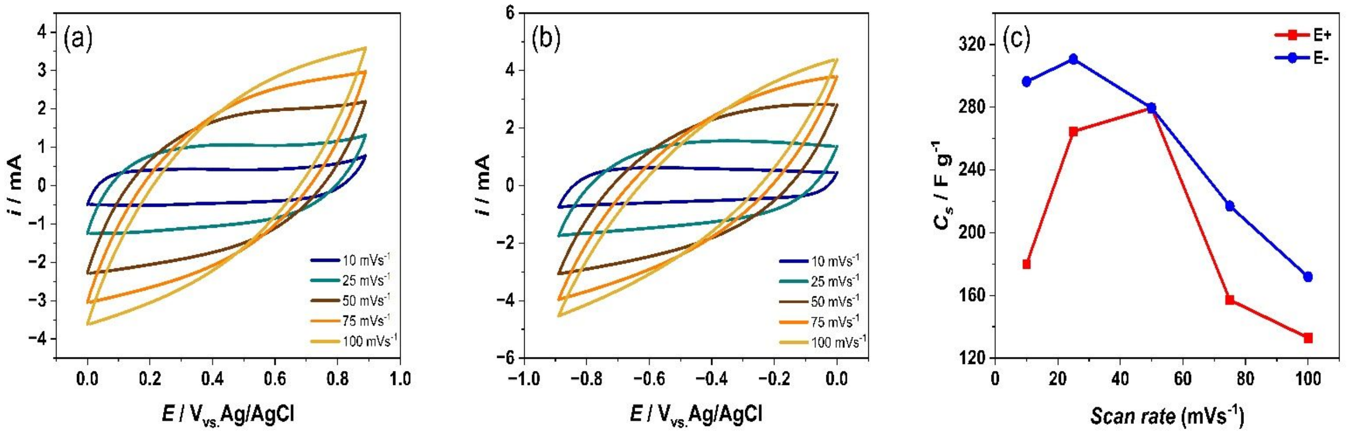

Figure S6: Electrochemical properties of 1 m LiTFSI on YEC-8A activated carbon, CVs from 10 mV s^{-1} to 100 mV s^{-1} (a) positive scan, (b) negative scan, and (c) specific capacitance vs. scan rates.

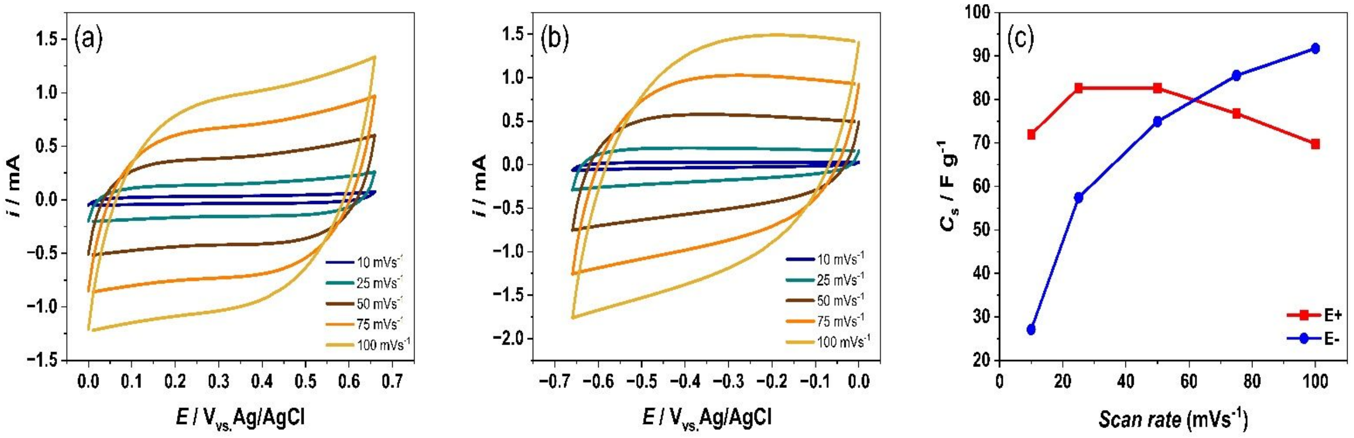

Figure S7: Electrochemical properties of 1 m Li_2SO_4 on YEC-8B activated carbon, CVs from 10 mV s^{-1} to 100 mV s^{-1} (a) positive scan, (b) negative scan, and (c) specific capacitance vs. scan rates

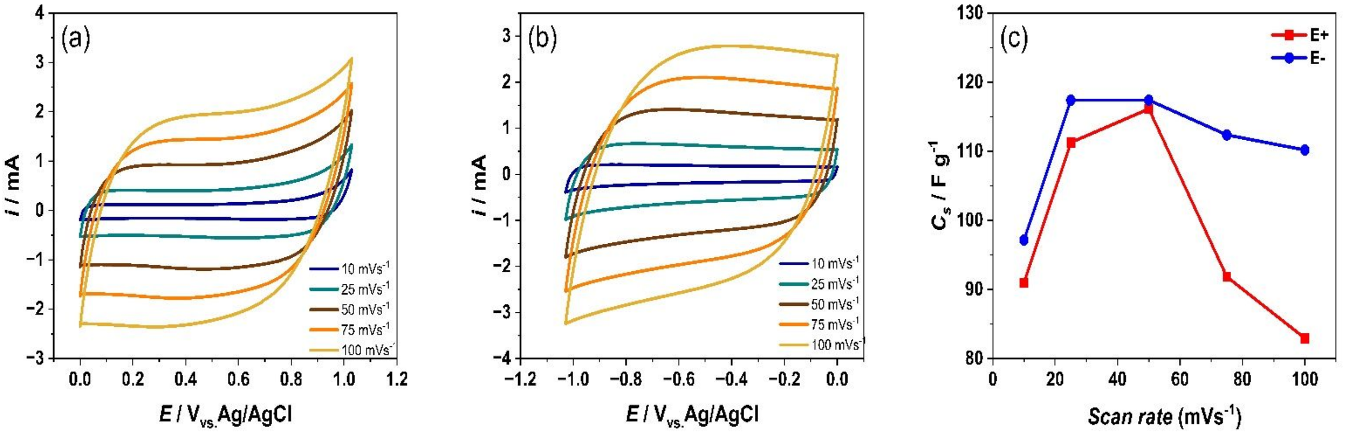

Figure S8: Electrochemical properties of 1 m LiCl on YEC-8B activated carbon, CVs from 10 mV s^{-1} to 100 mV s^{-1} (a) positive scan, (b) negative scan, and (c) specific capacitance vs. scan rates

Figure S9: Electrochemical properties of 1 m $LiNO_3$ on YEC-8B activated carbon, CVs from 10

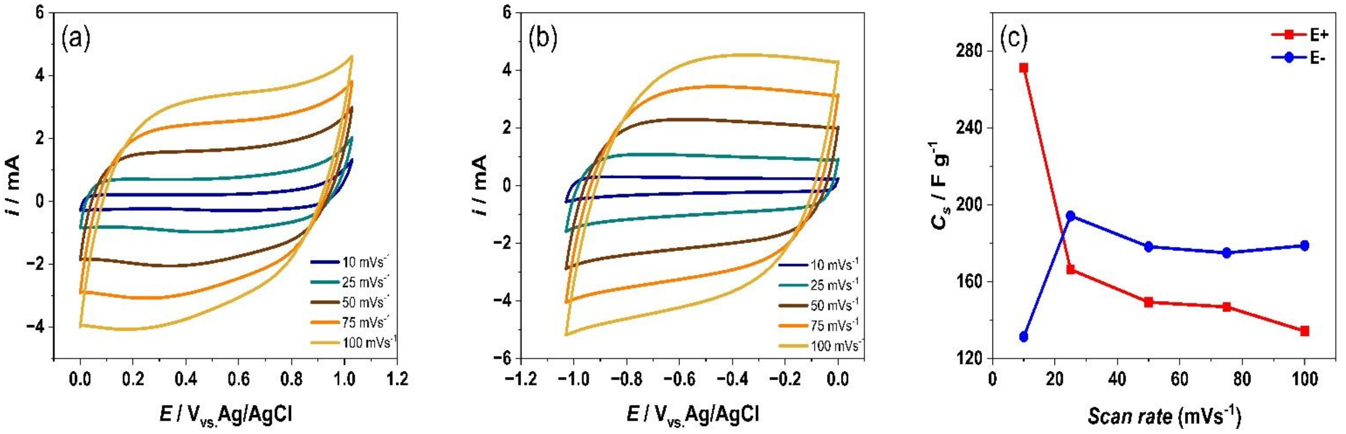

mV s^{-1} to 100 mV s^{-1} (a) positive scan, (b) negative scan, and (c) specific capacitance vs. scan rates

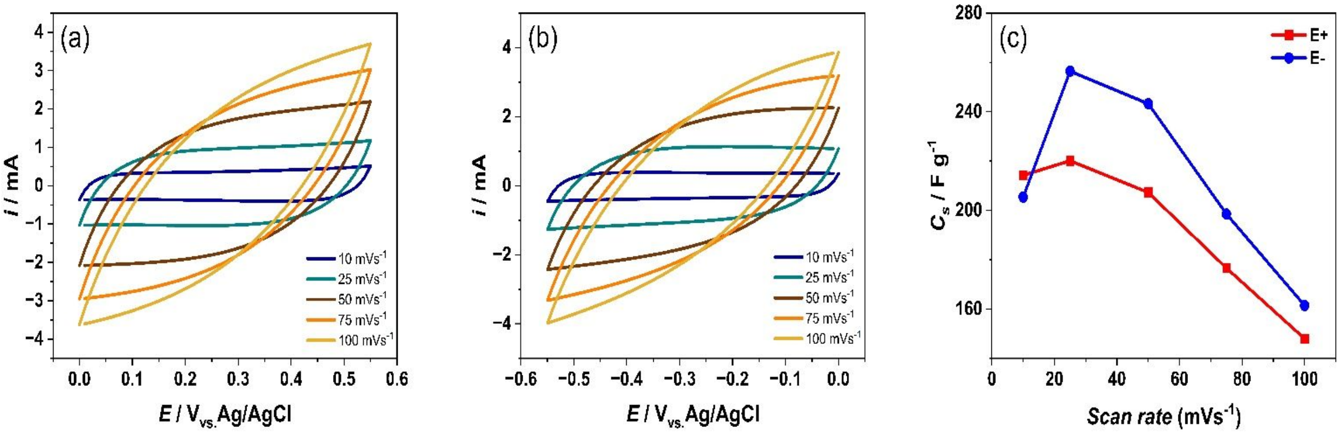

Figure S10: Electrochemical properties of 1 m $NaNO_3$ on YEC-8B activated carbon, CVs from 10 mV s^{-1} to 100 mV s^{-1} (a) positive scan, (b) negative scan, and (c) specific capacitance vs. scan rates

Note: the displayed capacitance value in (c) blue line is the specific capacitance calculated from negative branch window where red line represents the positive window.

Metrics evaluation

Table S.1 Metrics evaluation of the overall model in “Top half CV” and “Bottom half CV” model

DT model	MSE	MAE	MAPE
Top half CV (charge)	0.00128	0.01181	0.02339
Bottom half CV (Discharge)	0.69298	0.00876	0.02167

According to the prediction weight of the octic polynomial, the minimum and maximum coefficient of a polynomial equation is occur at x^8 and minimum of Top half CV and bottom half CV model are -1.6×10^7 and -1.8×10^7 respectively, The maximum of Top half CV model is 9×10^6 whereas The bottom half CV model is 1×10^7 this factor of a wide range of data could provide a poor value of R^2 after scaling process, It is advisable to use the Mean Absolute Percentage Error (MAPE) for a more accurate prediction assessment in percentage terms.

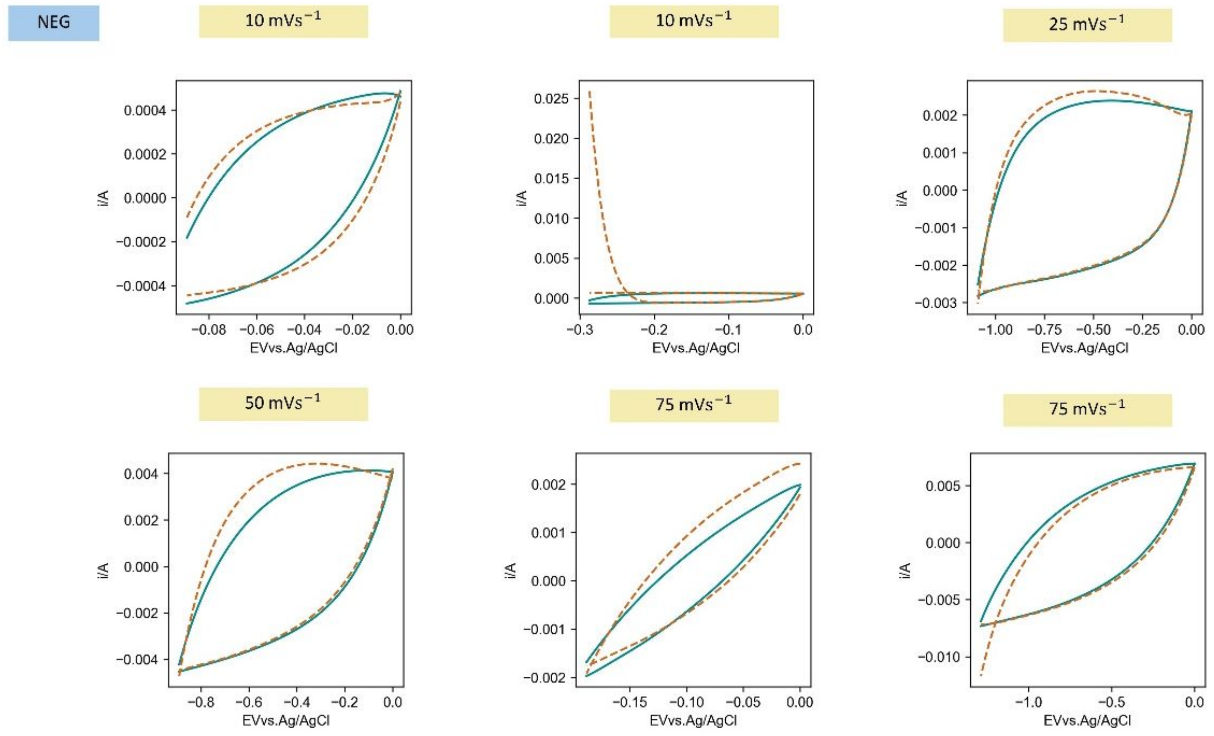

Figure S11: Comparison of CV profile at 1m LiTFSI in negative potential window with different scan rates and potential window. Top and bottom models are integrated; the dashed line represents the forecast, while the green line represents the experiment.

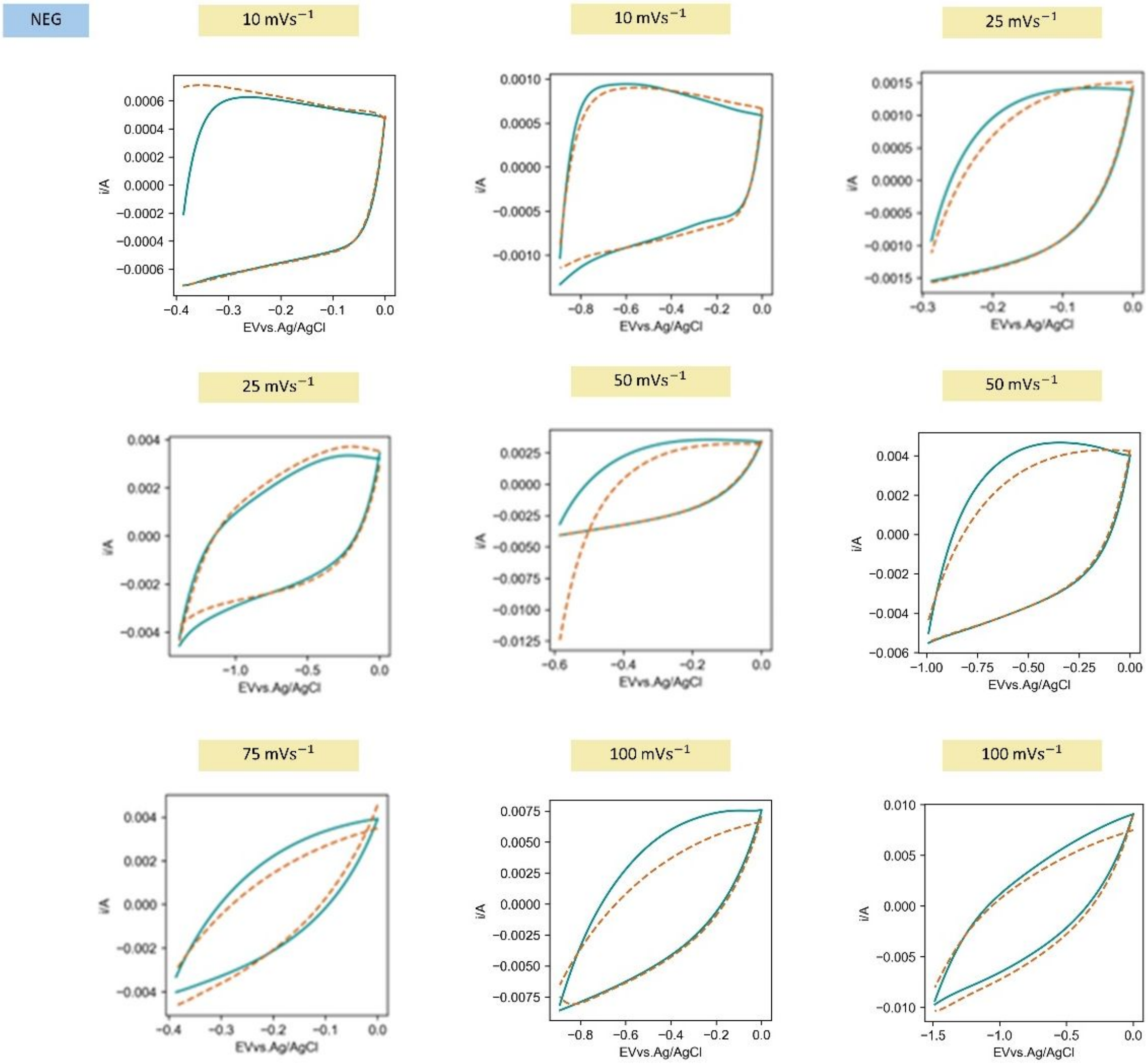

Figure S12: Comparison of CV profile at 5m LiTFSI in negative potential window with different scan rates and potential window. Top and bottom models are integrated; the dashed line represents the forecast, while the green line represents the experiment.

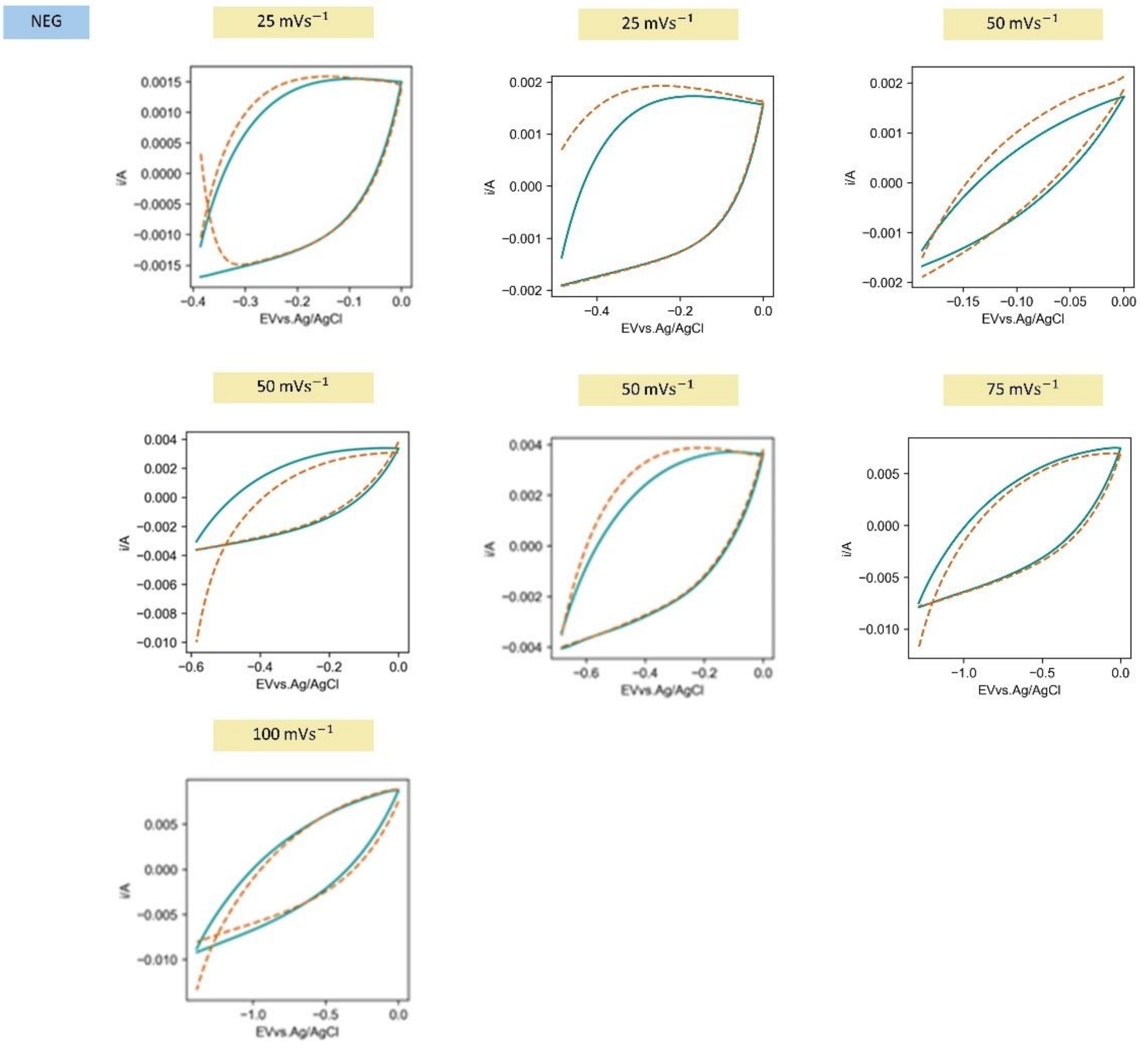

Figure S13: Comparison of CV profile at 10m LiTFSI in negative potential window with different scan rates and potential window. Top and bottom models are integrated; the dashed line represents the forecast, while the green line represents the experiment.

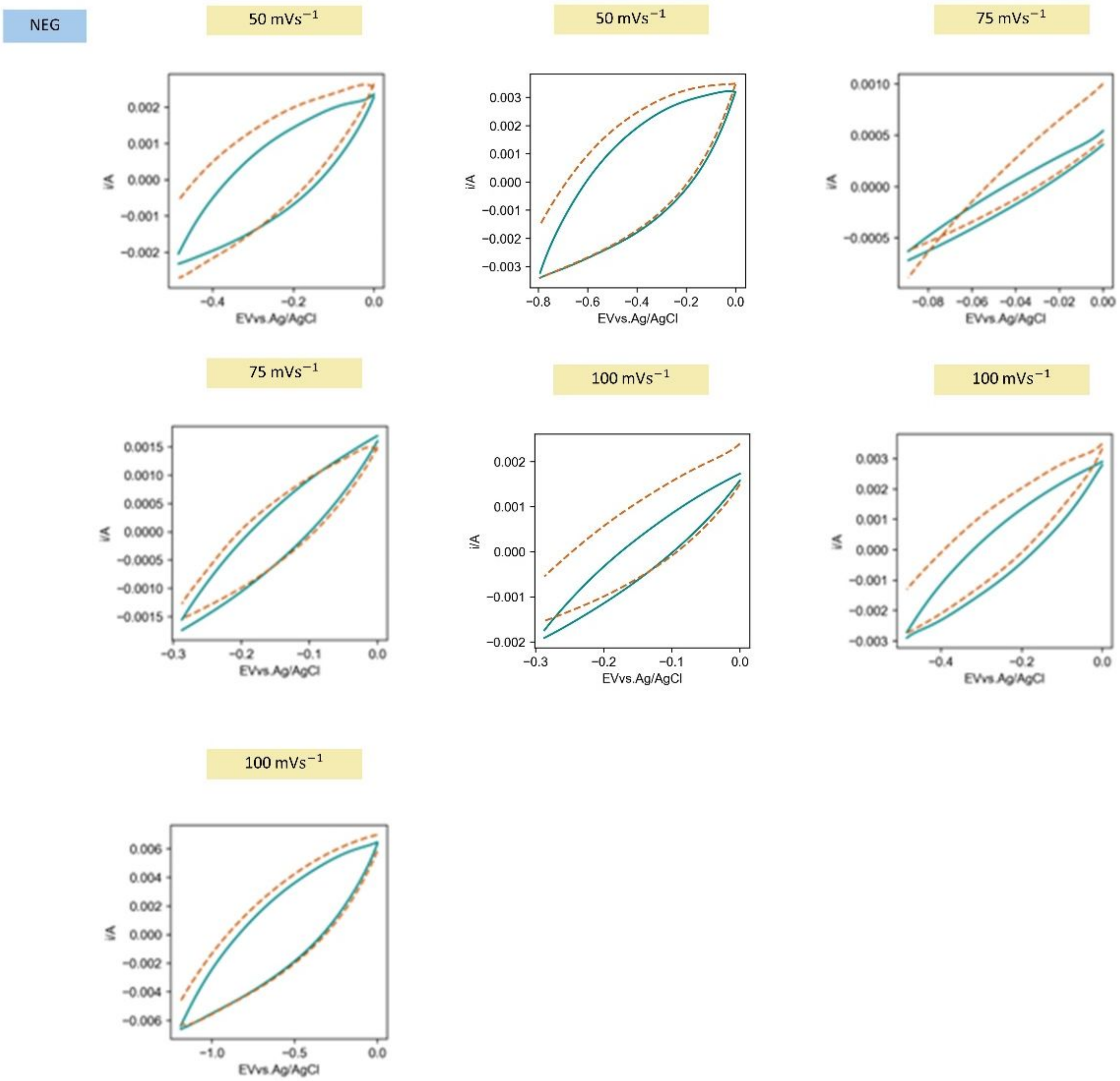

Figure S14: Comparison of CV profile at 20m LiTFSI in negative potential window with different scan rates and potential window. Top and bottom models are integrated; the dashed line represents the forecast, while the green line represents the experiment.

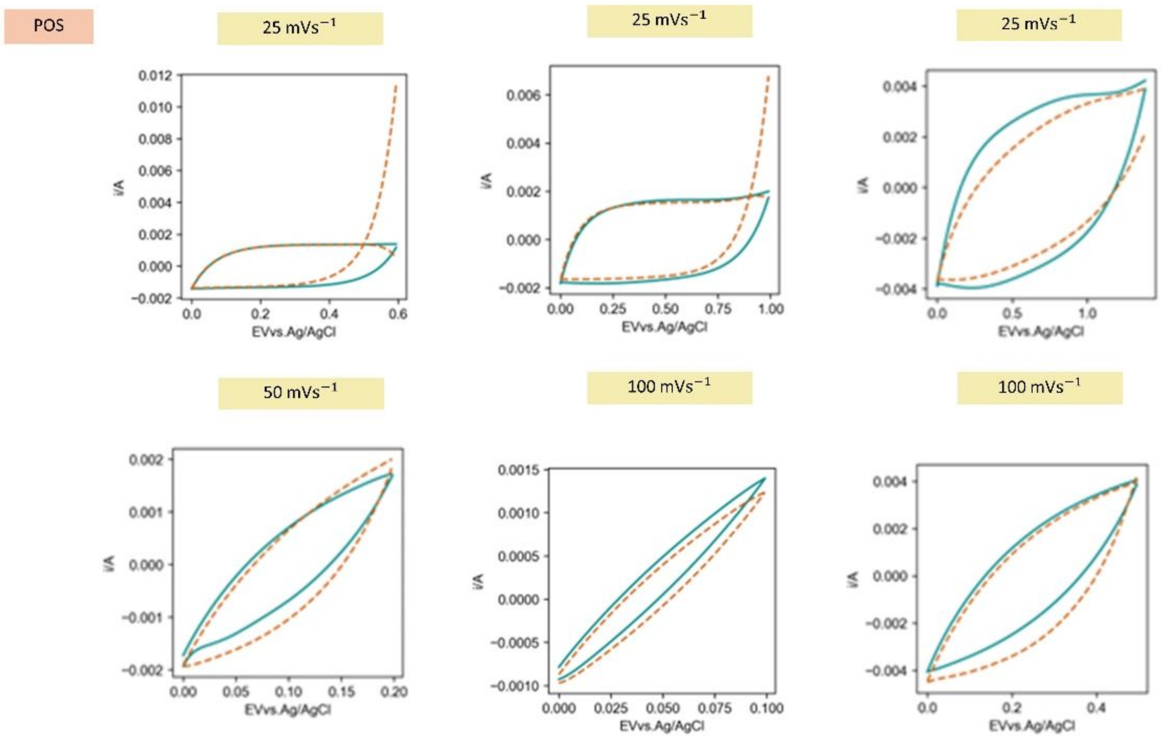

Figure S15: Comparison of CV profile at 1m LiTFSI in positive potential window with different scan rates and potential window. Top and bottom models are integrated; the dashed line represents the forecast, while the green line represents the experiment.

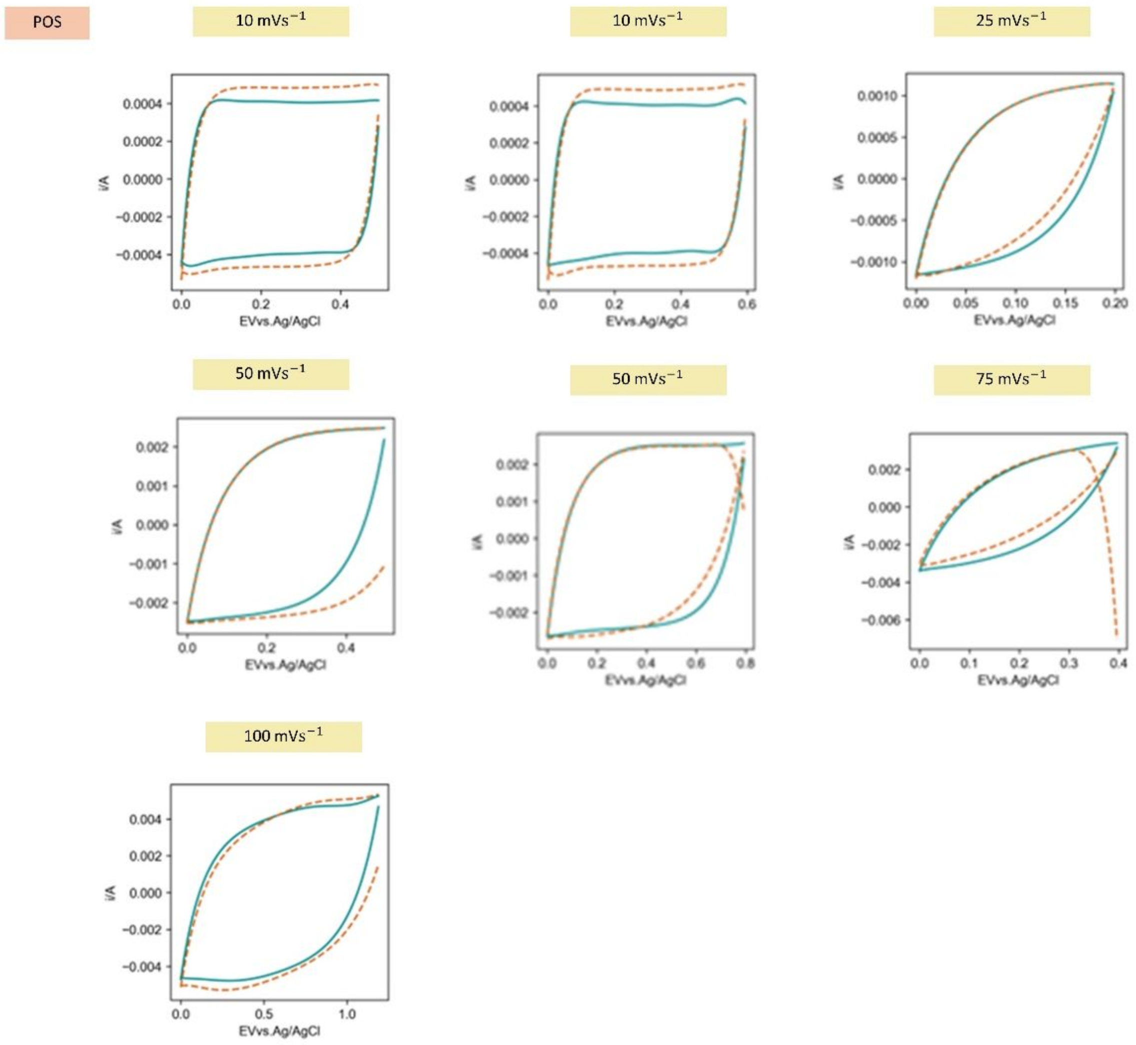

Figure S16: Comparison of CV profile at 5m LiTFSI in positive potential window with different scan rates and potential window. Top and bottom models are integrated; the dashed line represents the forecast, while the green line represents the experiment.

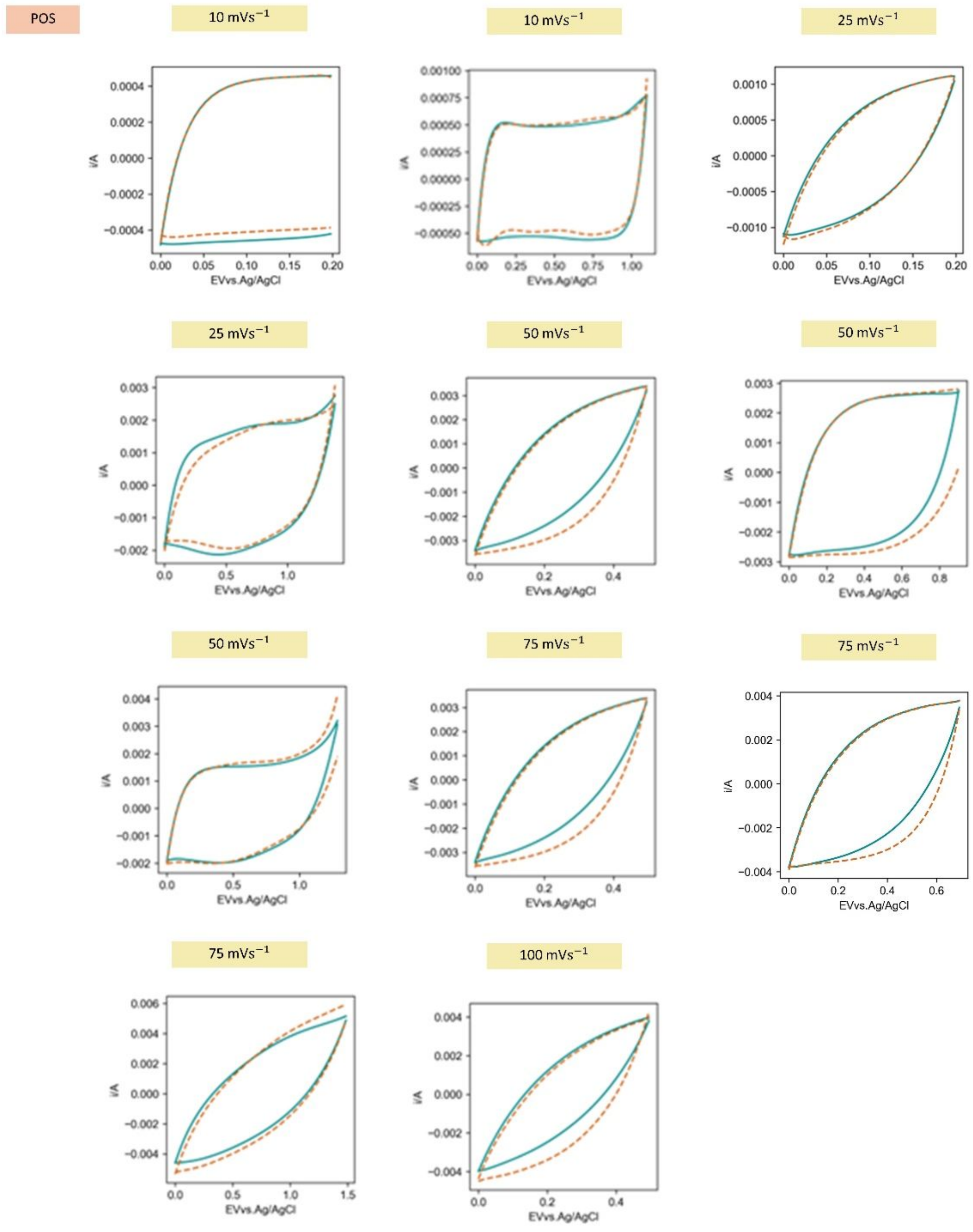

Figure S17: Comparison of CV profile at 10m LiTFSI in positive potential window with different scan rates and potential window. Top and bottom models are integrated; the dashed line represents the forecast, while the green line represents the experiment.

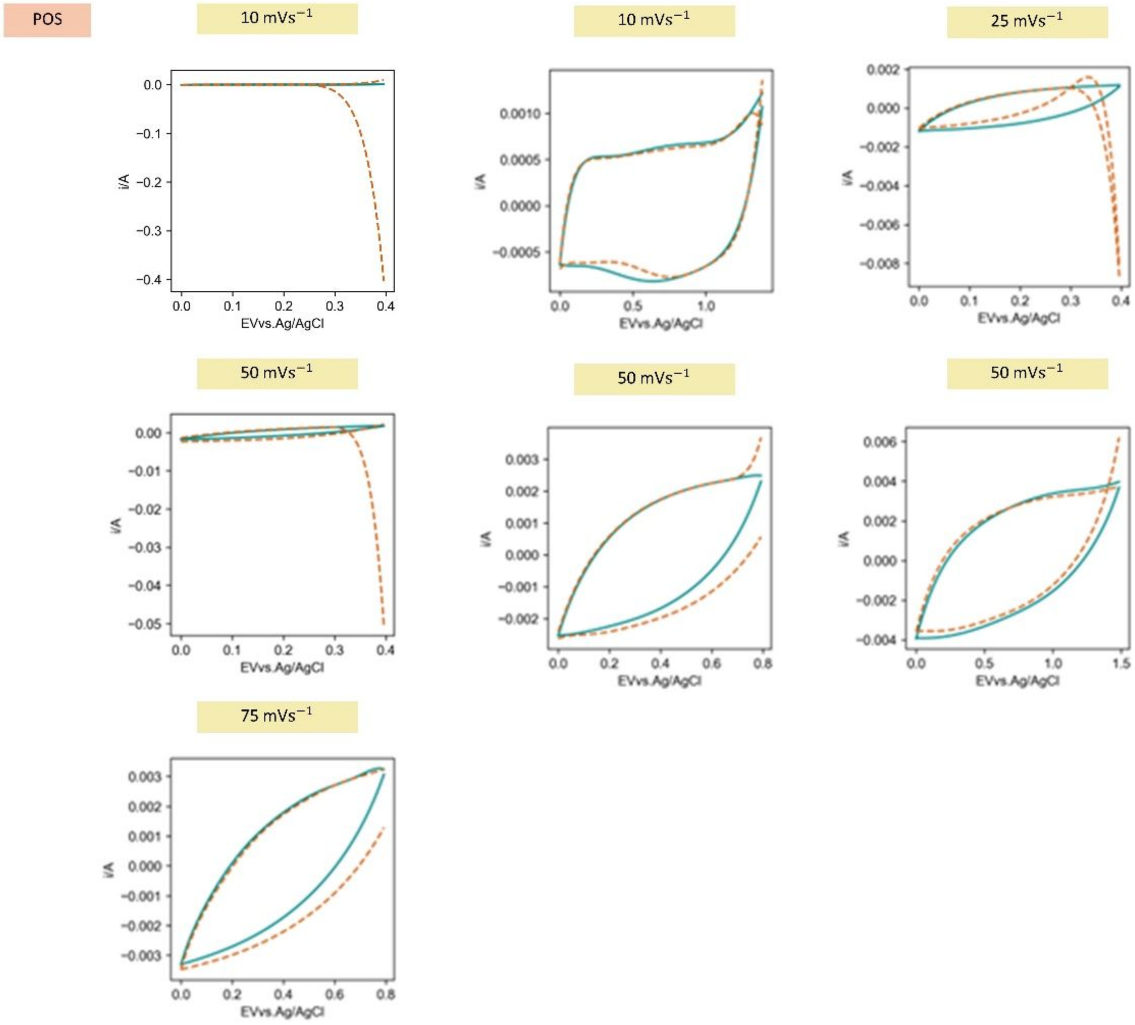

Figure S18: Comparison of CV profile at 20m LiTFSI in positive potential window with different scan rates and potential window. Top and bottom models are integrated; the dashed line represents the forecast, while the green line represents the experiment.

GitHub repository

Please use the link below to access our GitHub repository. The raw data, data preprocessing, and Decision tree model training are available in the repository. This work is under the GNU General Public License v3.0.

https://github.com/sklykor15/CVprediction_DT
